# *Bos taurus* (A-2) urine assisted bioactive cobalt oxide anchored ZnO: a novel nanoscale approach

**DOI:** 10.1038/s41598-022-19900-3

**Published:** 2022-09-16

**Authors:** Omkar S. Karvekar, Apurva S. Vadanagekar, Prashant D. Sarvalkar, Suresh S. Suryawanshi, Sarita M. Jadhav, Richa D. Singhan, Jyoti P. Jadhav, Kiran Kumar K. Sharma, Neeraj R. Prasad

**Affiliations:** 1 School of Nanoscience and Technology, Shivaji University, Kolhapur, MH 416004 India; 2 Department of Biochemistry, Shivaji University, Kolhapur, MH 416004 India

**Keywords:** Nanoscale materials, Nanobiotechnology, Other nanotechnology

## Abstract

In this study, a novel synthetic method for cobalt oxide (Co_3_O_4_) nanoparticles using *Bos taurus* (A-2) urine as a reducing agent was developed. In addition to this ZnO nanorods were produced hydrothermally and a nanocomposite is formed through a solid-state reaction. The synthesized materials were characterized through modern characterization techniques such as XRD, FE-SEM with EDS, DLS, zeta potential, FT-IR, Raman spectroscopic analysis, and TGA with DSC. The free radical destructive activity was determined using two different methods viz. ABTS and DPPH. The potential for BSA denaturation in vitro, which is measured in comparison to heat-induced denaturation of egg albumin and results in anti-inflammatory effects of nanomaterial was studied. All synthesized nanomaterials have excellent antibacterial properties, particularly against *Salmonella typhi* and *Staphylococcus aureus*. The composite exhibits excellent antioxidant and anti-inflammatory activities in comparison to pure nanomaterials. This reveals that these nanomaterials are advantageous in medicine and drug administration.

## Introduction

The scientific study of properties of materials and their applications at the nanoscale is the focus of nanoscience and technology. Richard Feynman, a renowned physicist and Nobel laureate, pioneered the concept of nanotechnology in 1959. Then, after extensive research took place in the domain of nanoscience and technology. In today’s world nanoscience and technology touches to diverse areas like electrochemical application^1^, sensors^2^, photocatalysis^3^, electronics^4^, Nano-catalyst in organic transformation reactions^5,6^, medical diagnosis^7^, cosmetics^8^, textile^9^, veterinary sciences^10^ and are becoming more attractive as novel uses, from medical diagnostics to gene therapy vehicles, drug delivery etc.

The properties of materials at the nanoscale differ from those in the bulk. This is due to the large surface area, inefficient gravitational forces, quantum state arrangement, unique free electron path and effective Van der Waals force of attraction. Due to exotic properties of materials at nanoscale and their world changer applications researchers from diverse fields were attracted to manufacture materials at nanoscale using several synthetic routes. The nanoscale materials can be synthesized through physical, chemical or biological approaches. The earlier attempts were made to synthesize nanomaterials through physical and chemical approaches. However, soon it was realized that these methods are restricted by complex processes that need the use of expensive instruments as well as toxic byproducts are formed^9,10^. Also, the nanomaterials produced through physical route of synthesis suffered from several defects, exposure of radiation. As a result, researchers from nanoscience and technology attempted to establish a cost-effective, eco-friendly alternative approach for producing defect-free nanomaterials which diminish the use of toxic chemicals. Thus, a group of researchers developed a biological approach for the synthesis of nanoscale materials. Biological methods of synthesis have grown in popularity among academics during the last 15 years. The researchers were able to effectively generate a large number of transition metal and their oxide nanoparticles using a biological method. In the earlier attempts researchers employing the biological way of synthesis used microorganisms such as fungus and bacteria, as well as plant extracts, to synthesize nanoscale materials^9,11^. The use of microorganisms to synthesize nanoparticles was expensive needed aseptic settings. The different parts of plants such as leaves, seeds, barks, fruits, roots, flowers are successfully used by the researchers for production of materials at nanoscale. The secondary metabolites present in the plants acts as natural capping agent and natural reducing agent. Soon it become very popular among the researchers due to its cost effectiveness and biocompatible nature. Our goal is to produce nanomaterials at the lowest possible cost and with the minimum resources available in the laboratory. The authors were inspired by Ayurveda, which is an ancient Indian holistic medical practice.

The ancient literatures in Ayurveda highly praises Indian cow urine and claims it has anti-neoplastic and anti-microbial properties. The antimicrobial property of Indian cow urine has been successfully examined in modern microbiological laboratories and observed to be really effective and as par with modern antibiotics^12^. According to laboratory examination, cow urine includes minerals such as iron, copper, nitrogen, manganese, silicon, magnesium, calcium salts, mineral salts, enzymes, and vitamins such as A, B, C, D, E, uric acid, and other hormones^6^. According to ancient Ayurvedic literature, cow urine can heal leprosy, peptic ulcers, liver illnesses, renal disorders, asthma, psoriasis, anemia, some types of allergies, abdominal distension due to accumulation of gases and cancer. The authors have first-hand knowledge of the laxative qualities of Indian cow urine^12^.

Thus, our research group has synthesized various transition metal and metal oxide NPs like cadmium oxide^13^, palladium^14^, copper oxide^15^, gold and silver^6^ using Indian cow urine. These nanoparticles, which were synthesized using Indian cow urine, have potent biological and catalytic effectiveness. Biological approaches of nanoscale synthesis based on Indian cow urine appear to be the most simple, cost-effective, fast, non-toxic, and ecologically friendly among all of the methods so far studied. According to modern knowledge in medicine, cobalt isotopes have anti-neoplastic properties. As a result, we attempted to combine the two, namely, cow urine for the production of cobalt oxide nanoparticles (Co_3_O_4_ NPs) and hydrothermally synthesized ZnO nanorods (NRs). Metal NPs and nanocomposites are attracting attention of the researchers from all over the world due to their superior electrical, magnetic, optical properties and biological capabilities. In this study, we have synthesized Co_3_O_4_ NPs using *Bos taurus indicus* urine and ZnO NRs using a hydrothermal approach and investigated their combined effect against various bacteria and fungi.

## Experimental section

### Materials

Ammonia Solution (30 wt%), Cetyl Trimethyl Ammonium Bromide (CTAB), Cobalt (II) Chloride Hexahydrate (CoCl_2_·6H_2_O), Zinc Nitrate Hexahydrate (Zn(NO_3_)_2_·6H_2_O) chemicals were procured from Sigma-Aldrich, Milwaukee (USA). With the agreement of the animal rearer, liquid metabolic waste from a cow farm in Kaneri village, Kolhapur, India (16.6237° N, 74.2722° E) was collected. The freshly discharged cow's liquid metabolic waste was collected in a clean screw-capped reagent bottle. Using Whatman filter paper (grade no. 3), the liquid metabolic waste from Gir cows was dribbled and stored at 5 °C for further experimentation.

Microbes namely *Escherichia coli* (NCLM2832), *Bacillus cereus* (NCLM2703), *Staphylococcus aureus* (NCLM2602), *Salmonella typhimurium* (NCLM2501), and Fungal strain *Aspergillus niger* (NCIM 1456) procured from the National Chemical Laboratory Pune were used for this study, However Strain *Fusarium solani* JALPK^16^ was collected from department of Microbiology, Shivaji University Kolhapur. The stock cultures were maintained on nutrient agar slants at 37 °C. All of the chemicals used in the experiments, including DPPH (2,2-diphenyl-1-picrylhydrazyl), ABTS (2,2′-azino-bis (3-ethyl benzothiazoline-6-sulfonic acid), and BSA (bovine serum albumin), were of analytical grade and purchased from Sigma (St Louis, MO, USA).

### Biomimetic-synthesis of Co_3_O_4_ NPs

Cobalt (II) Chloride was procured from Sigma Aldrich and used without further purification. Then we prepared 0.1 M CoCl_2_·6H_2_O solution in 100 mL of distilled water. To stabilize the NPs, a cationic surfactant, CTAB (0.1% w/v), was used. The solution was kept on a magnetic stirrer. Then cow urine was added dropwise in the beaker containing cobalt (II) chloride solution. After the addition of a sufficient amount of cow urine (i.e. 25 mL) in the cobalt (II) chloride solution, a whitish brown colored precipitate appeared in the solution.

*Bos taurus indicus* (A-2) urine contains urea and it acts as a reducing agent in this reaction. CTAB provides additional stability. When cow urine reacts with Co^2+^ ions, it gets converted into Co_3_O_4_. Thus, there is the formation of Co_3_O_4_ NPs. Once the Co_3_O_4_ nanomaterials were formed in the solution, it was centrifuged and the precipitate was separated and dried. Then the dried precipitate was kept in the furnace for about 2 h at 900 °C for annealing. The final product, which appeared dark blue, was collected and milled using the mortar and pestle into a fine granular powder. Lastly, the synthesized material was stored and used for further purposes like characterizations and applications.

### Plausible mechanism of action

**Figure 1:**
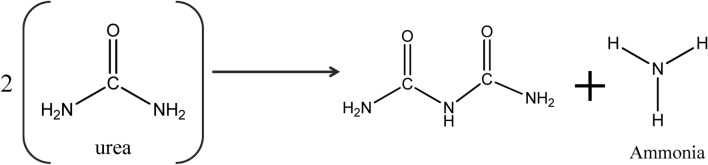
Possible mechanism of urea is converted to ammonia through hydrolysis.

The literature survey reveals that the liquid metabolic waste of *Bos taurus indicus* (A-2) contains urea, creatinine, aurum hydroxide, carbolic acid, phenol, calcium, and magnesium^6,12,17^. After photoactivation, a few biogenic volatile inorganics and organic compounds like CO_2_, NH_3_, CH_4,_ methanol, propanol, acetone, and some secondary nitrogenous products are also formed. The possible reaction mechanism for the Co_3_O_4_ NPs is given below:

The chemical formula of urea is CO(NH_2_)_2_. Here, the carbonyl group, i.e. C=O, is directly connected to two –NH_2_ groups. Actually, due to the presence of a lone pair of electrons on the nitrogen atom, urea seems to be a base. However, because of the electronegative character of the carbonyl group, it behaves as a neutral molecule. However, when urea is treated with the enzyme urease or at a high temperature, urea is converted to ammonia through hydrolysis. Urea is broken down into ammonia and isocyanate ions as a byproduct in the first step of the reaction. At pH levels of less than 5 and greater than 12, this reaction is reversible. Isocyanate is hydrolyzed to form ammonia in the second step (Shown in Fig. 1), and carbon dioxide is produced as a byproduct. The rate of urea hydrolysis is faster at 35 °C than at 15 °C. The pH impact is only noticeable between pH 6 and pH 8^6^.

### Hydrothermal synthesis of ZnO nanorods (NRs)

The morphology of zinc oxide nanomaterial depends upon its route of synthesis, the concentration of precursor, nature of the capping agent, reaction temperature, etc. ZnO NRs were synthesized through hydrothermal techniques. A 0.2 M zinc nitrate solution was prepared by dissolving the precursor in distilled water. In this experiment, 200 mL of zinc nitrate solution was taken in a 500 mL capacity borosilicate beaker. A magnetic needle was inserted into the beaker and kept on a magnetic stirrer. The magnetic needle was rotated at a high speed, around 700 rpm. Meanwhile, the burette was filled with 30 wt% ammonia solution, which was then dropped into the beaker while continuously stirring. Initially, a white precipitate was formed. However, after the addition of a few drops of ammonia, the solution turned colorless. When the solution became colorless, the ammonia addition was stopped, and the solution was transferred to an autoclave for hydrothermal treatment at 120 °C for 90 min. When the system was cooled to room temperature, the resultant product was collected and washed twice with distilled water followed by ethanol. In addition, the product was dried using the air-drying method at room temperature for 2 h, followed by a furnace for about 2 h at 450 °C for annealing. It was stored for further use^3^.

### Preparation of nanocomposites

At the end of the above experiments, we are getting cobalt oxide and zinc oxide in nano form. The nanocomposite can be synthesized by grinding these two nanomaterials with the help of the mechanical ball milling technique. For the synthesis of the nanocomposite, the desired proportion of Co_3_O_4_ and ZnO nanomaterials were taken in a mortar and finely grinded for about 3 h with a pestle. Here we employed the same method as used in mechanical ball milling or solid-state reaction. We tried to synthesize two composite materials with different proportions of Co_3_O_4_. We synthesized 5% w/w Co_3_O_4_ NPs with ZnO NRs Nano-composites (Co 5%) and 10% w/w Co_3_O_4_ NPs with ZnO NRs Nano-composite (Co 10%) materials. The mixture was put in the mortar and milled smoothly for about 3 h. In the synthesized composite material, there are no primary bonds like ionic, covalent, or metallic. Disparately due to secondary bonds like van der Waals, some weak attractive and repulsive forces are observed between Co_3_O_4_ NPs and ZnO NRs.

### Characterization study

After the successful synthesis of nanomaterials, it becomes very important to ensure their size, shape, surface charge, morphology, etc. As the materials at the nanoscale are beyond the perception of human eyes, we require advanced characterization techniques to reveal them.

The products were characterized by simultaneous thermal analysis, X-ray diffraction, scanning electron micrographs, etc. To illustrate the crystalline structure of the Co_3_O_4_ NPs, ZnO NRs, and Co_3_O_4_–ZnO nanocomposites, an X-ray diffractometer equipped with an irradiation line Kα of copper (Bruker D8 advanced, Germany) was used to record the XRD spectrum and their corresponding size was calculated using the Sherer equation. Furthermore, a Field Emission Scanning Electron Microscope (FESEM) is used to study the surface morphology of nanomaterials. Here, we used a TESCON MIRA-3 FESEM equipped with an Energy Dispersive Spectroscopy (EDS) detector to characterize Co_3_O_4_ NPs, ZnO NRs, and Co_3_O_4_–ZnO nanocomposites. The in-situ investigation of the interface was done using FT-IR, which revealed diverse functional groups adsorbed on the synthesized nanomaterials. The material was put on KBr pellets and we used an ALPHA Bruker FT-IR spectrometer. When using a Ranishaw Raman spectroscope, the Raman analysis ranges from 100 to 1000 cm^−1^. The particle size and charge were analyzed using Nano ZS 90 (Malvern, UK).

### The biomedical potential of nanoparticles

#### Minimal inhibitory concentration (MIC)

The antibacterial potential of synthesized Co_3_O_4_ NPs, ZnO NRs, and nanocomposites of both was evaluated by MICs^18^. The stocks of (1 mg/mL) nanomaterials were used after sonication, whereas different working solutions of nanomaterials (50, 100, 150, and 200 µg/mL) were made. In this experiment, new inoculums of gram-positive and gram-negative bacterial strains were used. All bacterial strains were inoculated individually in 100 mL of various nanomaterial concentrations and cultured for 20–24 h in a shaking incubator (REMI) at 100 rpm at 37 °C. Using a UV–Vis spectrophotometer, we evaluated the absorbance of each tube at 625 nm to see if microbial growth was inhibited or stimulated. A negative control as distilled water was maintained along with positive control of Streptomycin and Fluconazole (as standard reference substances) for antibacterial and antifungal potential, respectively, at a concentration of 1 mg/mL. MIC was defined as the lowest nanomaterial concentration at which the growth of bacterial cells was inhibited.

#### Agar well diffusion method for antibacterial and antifungal potential

The antibacterial and antifungal activities of the compounds are assessed using the standard agar well diffusion technique. With slight modifications, standard agar well diffusion procedures were used to determine the antibacterial efficiency of Co_3_O_4_ NPs and ZnO NRs against four distinct microorganisms^19^. 100 µL of different nanomaterial concentrations were placed into wells on agar plates, which were then kept at 4 °C for 30–40 min before being moved to an incubator for overnight storage at 37 °C. The plates were observed and the inhibitory zones were seen after 48 h. After this medium-range antibiotic, Streptomycin of a concentration of 100 µg/mL was used as a reference substance.

In similar consent, the PDA plates were prepared using submerged inoculation of two fungal strains: *Aspergillus niger* (NCIM 1456) and *Fusarium solani* JALPK^16^. The different concentrations of nanomaterials were introduced into the wells of PDA agar plates. These agar plates were then incubated at a temperature of 37 °C and the zone of inhibition was well measured in mm.

#### Antioxidant activity by ABTS and DPPH radical scavenging assay

Anti-oxidants are compounds having the potential to reduce the effects of free radicals produced by oxidative stress in the body, which are generated in various diseases. By performing the commonly used ABTS and DPPH methods, Co_3_O_4_ NPs prepared from cow urine and hydrothermally synthesized ZnO NRs are analyzed for their antioxidant potential by performing the commonly used ABTS and DPPH methods. The ABTS radical scavenging assay was performed as described by Re et al.^20^ with a few modifications^21^. 50 μL of nanomaterial water extract with 1 μg/mL of concentration was mixed with 2950 μL of ABTS reagent. Thereafter, the absorbance of the aliquot was measured after 2 h of incubation under dark conditions and measured at 734 nm to produce a sample. To produce a control, methanol (99.5%) was used as a blank solution and its absorbance was recorded. Positive control was ascorbic acid.

The DPPH assay was carried out using the method as described by Brand Williams^22^ with slight modification^23^. The reaction was done by mixing 40 μL (1 μg/mL) nanomaterials with 3 mL of DPPH reagent, and incubation was done by avoiding light oxidation for 30 min. The spectrophotometric absorbance at 517 nm was used to determine the decrease of the DPPH radical. The radical-scavenging activity (RSA) is calculated using Eq. (1),

1 $$RSA\;(\%) = \frac{{{\text{Acontrol}}- {\text{Asample}}}}{{{\text{Acontrol}}}} \times 100$$

where Asample is the absorbance of the solution when the sample has been added at a particular level, Acontrol is the absorbance of the DPPH or ABTS solution.

### In vitro assays for anti-inflammatory study

#### Protein denaturation assay

The denaturation of protein causes changes in the physiochemical properties of the protein, which are caused by inflammatory agents. This method is explained by Grant et al.^24^ with slight modification^25^. The 50 μL nanomaterials working solution was diluted with 450 μL of 5% w/v BSA before being incubated at 37 °C for 20 min and heated at 57 °C for 3 min. Tubes were cooled under running water and diluted with 2.5 mL phosphate buffer saline and absorbance was measured at 660 nm. The percent of inhibition of the Fetal Bovine Serum (BSA) protein is calculated by Eq. (2),

2 $${\text{BSA}}\;{\text{Denaturation}}\;{\text{inhibition }}\;({{\%}}) = \frac{{{\text{Acontrol}}-{\text{Asample}}}}{{{\text{Acontrol}}}} \times 100$$

where Acontrol is absorbance of the control, Asample is the absorbance of the test sample.

#### Leukocyte membrane stabilization test

This experiment is depending on hypotonicity-induced hemolysis of human red blood cells (HRBC) and the measurement of Hemoglobin content was measured at 560 nm. The experiment was described by Bhurat et al.^26^ with the small modification described^27^. This study used diclofenac (50 µg/mL) as standard medicine. Measurements of percent stabilization of Leukocytes [Eq. (3)] by considering control as 100%.

3 $${\mathrm{RBC}}\,{\text{Stabilization}}\,{\text{leukocyte}}\,({\%}) =100-\left(\frac{\mathrm{Asample}}{\mathrm{Acontrol}}\right)\times 100$$

where Acontrol is the absorbance of the control, Asample is the absorbance of the test sample.

#### Statistical analysis

Experiments were set up in a completely randomized block design and each experiment was repeated thrice. Results were expressed as Mean ± SD. Statistical analysis was carried out using Graph Pad Prism 5 software. The significance of the experiment was estimated by determining the *p* value (*p* < 0.05) by one-way ANOVA Dunnett's multiple comparison test.

## Results and discussion

### XRD study

**Figure 2:**
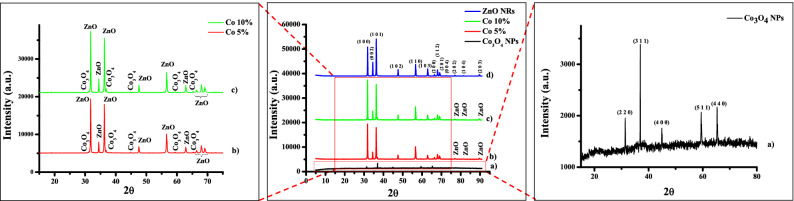
XRD pattern of (**a**) Co_3_O_4_ NPs, (**b**) Co 5% nanocomposite, (**c**) Co 10% nanocomposite and (**d**) ZnO NRs.

The poly-dispersed crystalline nano-material is revealed by the XRD spectrum. The XRD patterns of Co_3_O_4_ NPs, ZnO NRs, and their composites are shown in Fig. 2. The peak position denotes the unit cell's translational symmetry, i.e., its size and shape, whereas the peak intensities denote the electron density within the unit cell. Co_3_O_4_ NPs show Bragg's reflections, as shown in Fig. 2a, (enlarged spectra on the right side) with 2θ values at 31.382, 36.9599, 44.8974, 59.4242, and 65.3031 representing (220), (311), (400), (511), and (440) planes, respectively; these planes are due to the cubic Nanocrystalline structure of Co_3_O_4_ NPs. The pattern was dependable, as evidenced by card No. 01-076-1802 in the JCPDS database^28^.

The spectra of ZnO NRs (Fig. 2d) correspond to JCPDS card No. 03-065-3411, confirming the hexagonal wurtzite-type structure. The observed 2θ values of ZnO NRs are: 31.8383, 34.4921, 36.3209, 47.5976, 56.6386, 62.8961, 66.4211, 67.9848, 69.1256, 72.6216, 76.992, 81.4166, and 89.631, which correspond to the (h k l) values (100), (002), (101), (102), (110), (103), (200), (112 (203). The JCPDS cards show that the synthesized nanomaterials are entirely crystalline, with no adulterations^29–31^. The spectra of Co 5% and Co 10% (Fig. 2b,c) give the conformation of the synthesis of nanomaterials. The spectrum corresponds to JCPDS cards 01-076-1802 (Co_3_O_4_ NPs) and 03-065-3411 (ZnO NRs) (enlarged spectrum on right the side).

The Debye–Scherer’s formula [Eq. (4)] was used to compute the crystallite size of the nanomaterials from the Full Width at Half Maxima (FWHM) denoted by β and the Diffraction angle (θ),

4 $$D= \frac{0.9\uplambda }{{\upbeta \cos\uptheta }}$$

here in Eq. (4) where λ is the wavelength of the X-ray used for diffraction (0.1540 nm).

The crystallite size for various samples is calculated using the above formula and is represented in Table 1 for Co_3_O_4_NPs and in Table 2 for ZnO NRs.

**Table 1:** XRD analysis and calculation of various parameters for Co_3_O_4_ NPs.

Sr. No.	Peak position: 2θ (°)	Full-width half maxima (°)	Full-width half maxima (radians)	Particle size D (nm)	d-spacing (Å)	For Ag unit cell edges: a = b = c (Å)	Specific surface area (m^2^/g)	Morpho-logical indexing
1	31.382	0.121	0.002111	68.2337	2.85058	8.0720	14.51041	0.666743
2	36.9599	0.1614	0.002816	51.9254	2.43219		20.41525	0.599983
3	44.8974	0.2421	0.004223	35.5234	2.01892		29.84148	0.499982
4	59.4242	0.1614	0.002816	371.388	1.55543		2.854346	0.599983
5	65.3031	0.1476	0.002575	63.9629	1.42773		16.5732	0.62123

**Table 2:** XRD analysis and calculation of various parameters for ZnO NRs.

Sr. No.	Peak position: 2θ (°)	Full-width half maxima (°)	Full-width half maxima (radians)	Particle Size D (nm)	d-spacing (Å)	For Ag unit cell edges: a = b ≠ c (Å)	Specific surface area (m^2^/g)	Morpho-logical indexing
1	31.8383	0.2017	0.003519	40.98028	2.81076	a = b = 3.2495 c = 5.2069	25.86782	0.545498
2	34.4921	0.2017	0.003519	41.26349	2.60034		25.69028	0.545498
3	36.3209	0.1614	0.002816	51.83253	2.4735		20.45184	0.599983
4	47.5976	0.2421	0.004223	35.8845	1.9105		29.54119	0.499982
5	56.6386	0.2017	0.003519	44.76501	1.62513		23.68079	0.545498
6	62.8961	0.2017	0.003519	46.19228	1.47767		22.94909	0.545498
7	66.4211	0.1614	0.002816	58.86456	1.40755		18.00864	0.599983
8	67.9848	0.1614	0.002816	59.39704	1.37893		17.8472	0.599983
9	69.1256	0.1614	0.002816	59.80096	1.35894		17.72665	0.599983
10	72.6216	0.1614	0.002816	61.11453	1.3019		17.34564	0.599983
11	76.992	0.121	0.002111	83.93634	1.23853		12.62946	0.666743
12	81.4166	0.1614	0.002816	64.96845	1.18204		16.3167	0.599983
13	89.631	0.1614	0.002816	69.42914	1.0938		15.26838	0.599983

#### Morphology Index (MI)

The interrelation between particle size and morphology determines the specific surface area of NPs. FWHM is used to determine MI. MI is calculated using the following Eq. (5),

5 $$\mathrm{MI}=\frac{{FWHM}_{h}}{{FWHM}_{h} + {FWHM}_{p}}$$

The particulate FWHM value of a peak is FWHM_p_, and FWHM_h_ is the highest FWHM value obtained from peaks.

**Figure 3:**
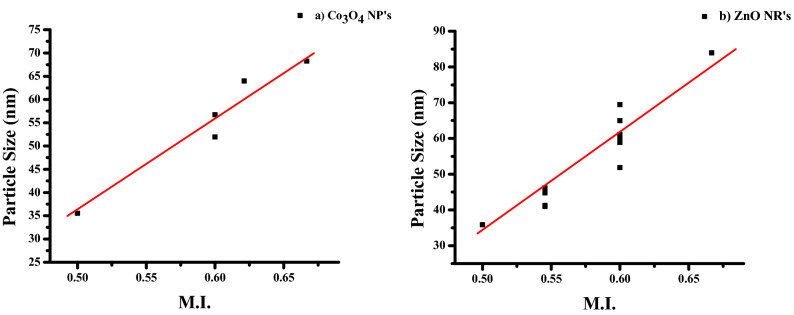
Morphological Index versus particle size of (**a**) Co_3_O_4_NPs and (**b**) ZnO NRs.

**Figure 4:**
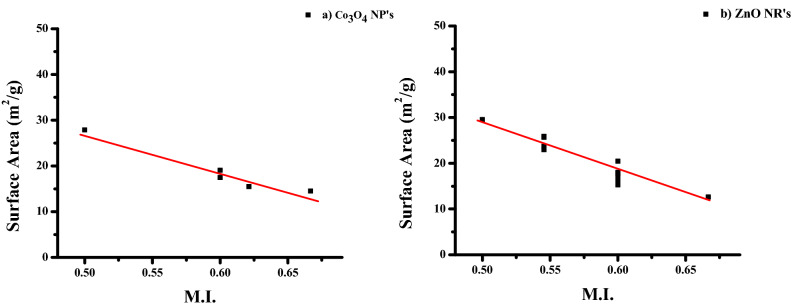
Morphological Index versus specific surface area of (**a**) Co_3_O_4_NPs and (**b**) ZnO NRs.

The MI value of Co_3_O_4_ NPs ranges from 0.4998 to 0.6667 (Table 1), whereas the estimated values of ZnO NRs also range from 0.4998 to 0.6667 (Table 2). It is linked to the size of crystalline particles and the Specific Surface Area (SSA). Table 1 shows that Co_3_O_4_ NPs have an SSA value of 2.8543–29.8414 m^2^/g (Table 1), while ZnO NRs have an SSA value of 12.6294–29.5411 m^2^/g (Table 2). According to the estimated data, MI is directly proportional to particle size and inversely proportional to SSA with a minor fluctuation. Figures 3a,b and 4a,b show the results. The linear fit indicates the deviations and relationships between the figures.

#### Surface morphology

**Figure 5:**
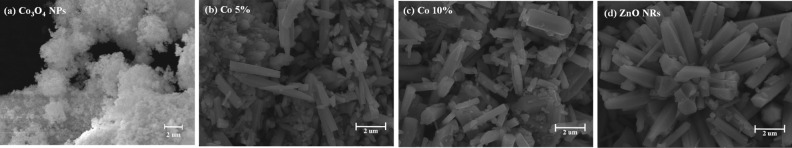
FE-SEM images of (**a**) Co_3_O_4_ NPs, (**b**) Co 5% nanocomposite, (**c**) Co 10% nanocomposite and (**d**) ZnO NRs.

**Figure 6:**
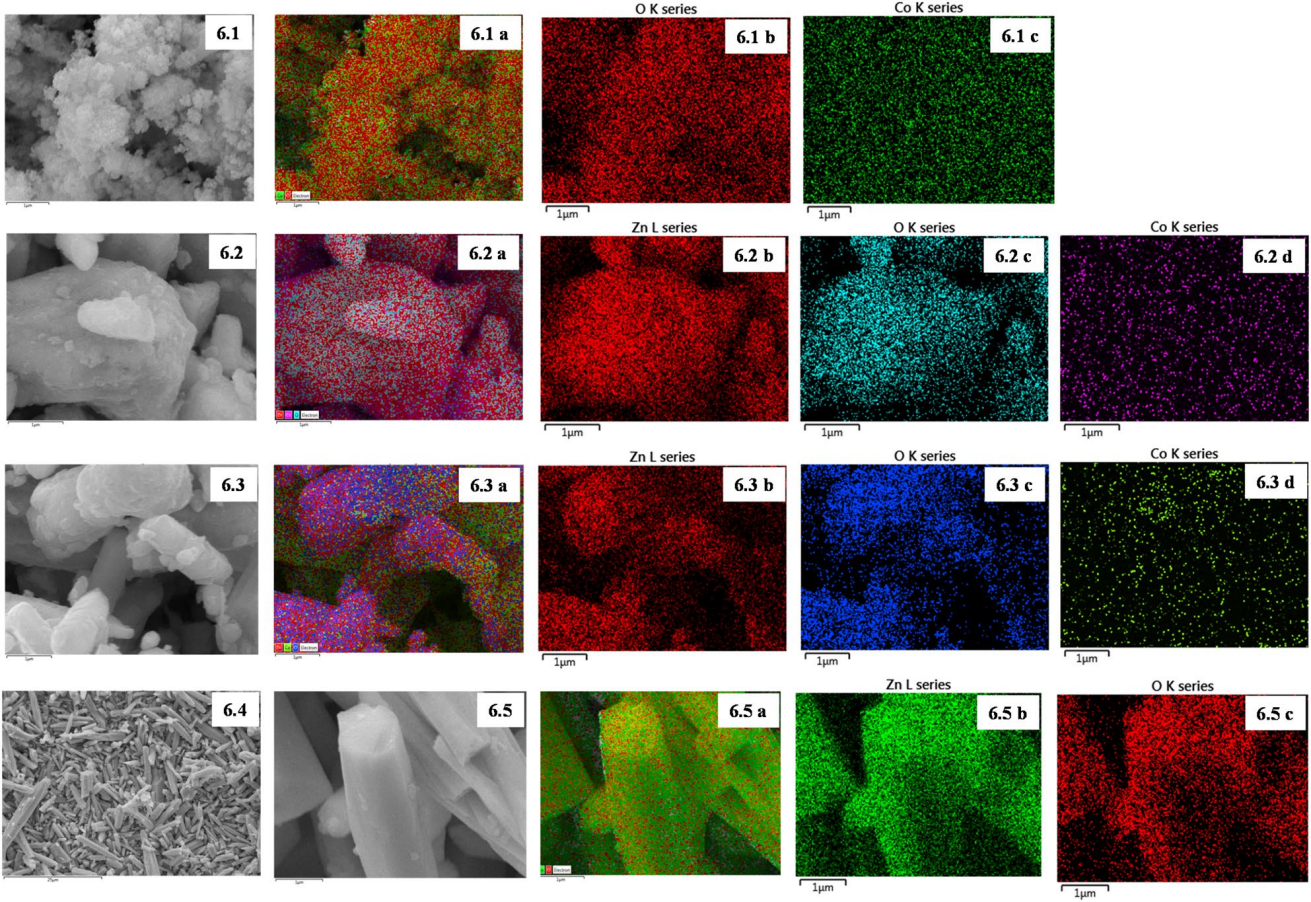
Demonstrates the data of elemental mapping. (**6.1a**, **6.2a**, **6.3a**, **6.5a**) Merged image of O, Co, and ZnO. (**6.1b–c**, **6.2b–d**, **6.3b–d**, **6.5b–c**) Elemental mapping images of O, Co, and ZnO elements of Co_3_O_4_ NPs, Co 5%, Co 10% and ZnO NRs, respectively.

FE-SEM was used to study the shape and size of synthesized nanomaterials and their composites (Fig. 5). Furthermore, looking at the low magnification shows that particles grown at a high density are relatively spherical shaped. On further magnification, it reveals that Co_3_O_4_ NPs tend to agglomerate. Most of the synthesized Co_3_O_4_ NPs lie in the size range of 100–400 nm. (Fig. 5a). Apart from the major spherical shape, some irregularly shaped nanomaterials are observed in FE-SEM imaging^32^. The FESEM image (Fig. 5b,c) of the synthesized nanocomposite flashes the formation of Co_3_O_4_ NPs anchored on the surface of the ZnO NRs. Speculation of the image reveals that the ZnO NRs are well aligned and the Co_3_O_4_ NPs are randomly dispersed in them. The FESEM images of the NRs are shown in Fig. 5d. The FESEM image reveals that the diameters of the ZnO NRs lie in the range of 600–1000 nm, and the length of the nanorod is in the range of 2000–3000 nm^33^. Furthermore, Fig. 6 also shows the elemental mapping of Co_3_O_4_ NPs, ZnO NRs, and their composites. Firstly, in Fig. 6.1a–c,6.2a–d,6.3a–d,6.5a–c, we can see the combined mapping of elements. Nevertheless, Fig. 6 consists of individual mappings of the element’s cobalt, oxygen, and zinc.

#### Energy dispersive spectroscopy (EDS) analysis

**Figure 7:**
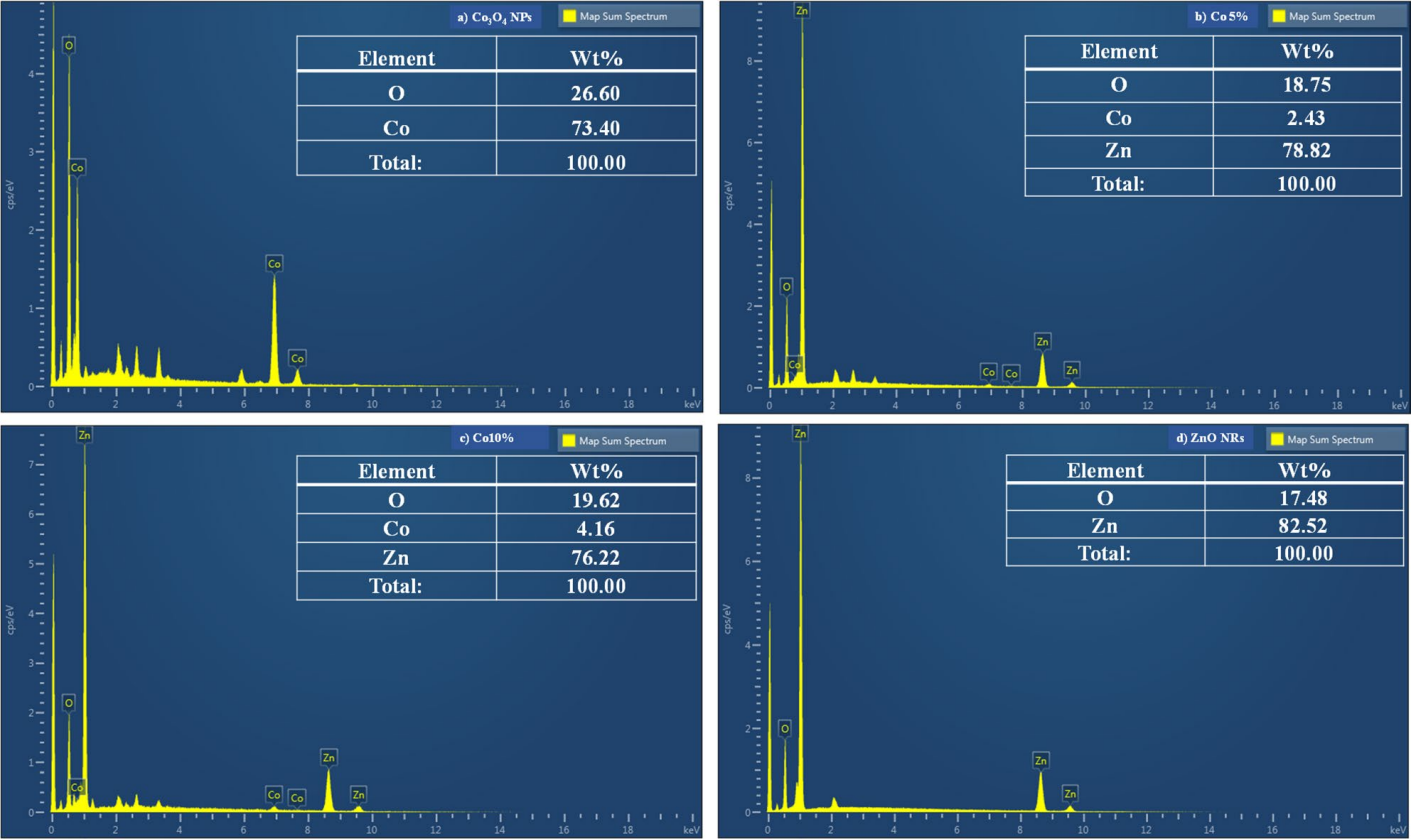
EDS analysis of (**a**) Co_3_O_4_ NPs, (**b**) Co 5% nanocomposite, (**c**) Co 10% nanocomposite and (**d**) ZnO NRs.

Elemental composition analysis of synthesized nanomaterials and their composites has been studied through the Energy Dispersive (ED) spectra. The characteristic ED spectra are shown in Fig. 7 and the analysis results are summarized in the table. In the spectrum of Co_3_O_4_NPs (Fig. 7a), four peaks are observed, which are identified as cobalt and oxygen. However, in the spectrum of ZnO NRs (Fig. 7d), there are also 4 peaks with Zinc and oxygen^34^. Even the traces of impurities and other elements are not observed. The observed composition ratios of Co_3_O_4_ and ZnO in the composite are consistent with the expected composition ratio and are shown in Fig. 7b,c. This indicates that the expected stoichiometry under preparation is well maintained in the samples prepared using the mortar-pestle^35^.

To explain the composition of the material in wt%, in Co_3_O_4_NPs, 73.40% of the total weight is cobalt and the remaining is oxygen. Moreover, talking about composites in Co 5% and Co 10%, the largest portion of the total weight is acquired by zinc, i.e., 78.82% and 76.22%, respectively. However, the smallest portion is made up of cobalt, which contains 2.43% and 4.16%, not to mention, that the remaining oxygen is out of total weight. There is 17.48% oxygen and 82.52% zinc in ZnO NRs.

#### FT-IR analysis

**Figure 8:**
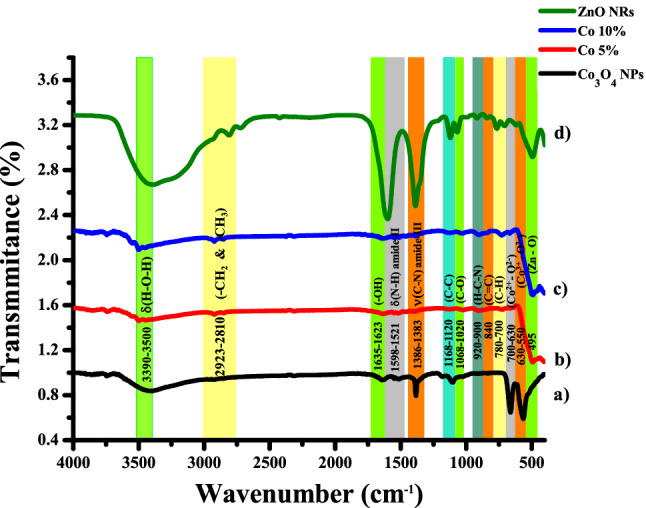
FTIR spectra of (**a**) Co_3_O_4_ NPs, (**b**) Co 5%, (**c**) Co 10% and (**d**) ZnO NRs.

The FT-IR spectra of ZnO, Co_3_O_4,_ and ZnO–Co_3_O_4_ Nano-composites were recorded in the range of 4000 to 400 cm^−1^. The plot (Fig. 8) illustrates the FT-IR spectrum of the Co_3_O_4_ NPs, Co 5%, Co 10% nanocomposite, and ZnO NRs. Overall, this spectrum gives information about different types of vibrations in these samples. The data of Co_3_O_4_ NPs shown in Fig. 8a is the fingerprint of Co_3_O_4_. The spectrum of Co_3_O_4_ NPs, Co 5%, Co 10% and ZnO NRs, a broad peak centered at 3395, 3498, 3500, and 3390 cm^−1^ is because of δ(H–O–H)^36^. In Fig. 8b,c, the Co 5% and Co 10% asymmetric stretching vibration –CH_3_ and –CH_2_ groups absorption bands, respectively, were observed at 2922 cm^−1^ and 2923 cm^−1^ while the ZnO stretching vibration of the –CH_2_ group is at 2810 cm^−1^ and it is illustrated in Fig. 8d. However, –OH groups of water molecules are responsible for a small peak centered at 1629, 1635 and 1623 cm^−1^ in spectrum of all samples except ZnO nanorods. Furthermore, these peaks show the presence of humidity. Besides, this δN-H (amide II) group is confirmed due to the presence of significant peaks at 1530, 1521, and 1598 cm^−1^ in Co_3_O_4_, Co 5%, and ZnO NRs, respectively. In the same way, the transmittance band at 1383, 1385, and 1386 cm^−1^ in Co 5%, Co 10%, and ZnO NRs resulted in the presence of group νC-N (amide III). Moreover, the band at 1103 cm^−1^ in Co_3_O_4_NPs, and 1168 (for Co 5% & 10%) and 1120 cm^−1^ in ZnO NRs occurs because of the stretching vibrations of C–C linkages correspondingly^37^. The stretching vibrations of C–O stretching cause the band to appear at 1068–1020 cm^−1^ in Co 5%, Co 10%, and ZnO. The bands between 900 and 920 cm^−1^ are due to H–C–N functional group. The peak at 840 cm^−1^ in ZnO shows there is C=C bending. The peak ranges from 780 to 700 cm^−1^ due to C–H bending. Nevertheless, the peak in the 700–630 cm^−1^ range accounts for Co^2+^–O^2−^ in tetrahedral coordination, and the peak in the 630–550 cm^−1^ range stands for Co^3+^–O^2−^ in octahedral coordination^38^. The transmittance peak at 495 cm^−1^ is likewise ascribed to Zn–O vibrations^39^.

#### Raman spectroscopic analysis

**Figure 9:**
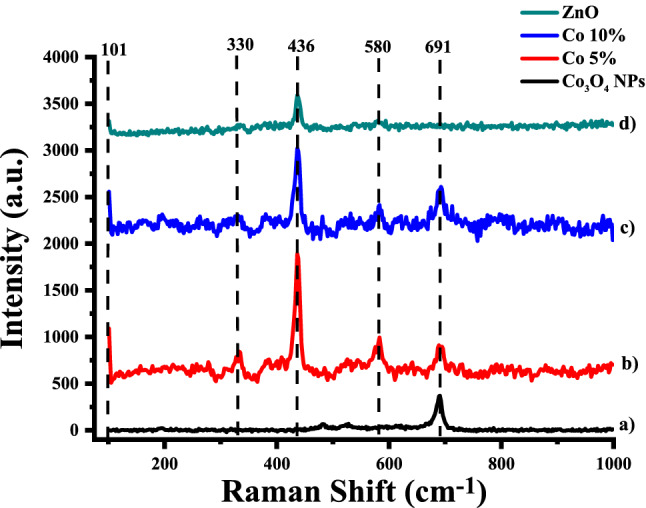
Raman spectra of (**a**) Co_3_O_4_ NPs, (**b**) Co 5%, (**c**) Co 10% and (**d**) ZnO NRs.

The optical properties of as-synthesized Co_3_O_4_ NPs, ZnO NRs and their composites were characterized using Raman spectroscopy. The presence of defects was detected using Raman spectroscopy, which was utilized to detect the disorder caused by dopant incorporation in the host lattice. Figure 9 illustrates the Raman Spectra of Co_3_O_4_ NPs, Co 5%, Co 10% Nanocomposite, and ZnO NRs samples taken at RT in the range of 100–1000 cm^−1^. It is observed that in Fig. 9a,b,c there is a common peak at 691 cm^−1^, which is because of the A_1_g phonon mode of Co_3_O_4_. The peak at 580 cm^−1^ is attributed to the B1 (high) phonon and the peak at 380 cm^−1^ is attributed to the A1 (TO) mode in the ZnO, Co 5% and Co 10%^40^. However, there are two common peaks in Fig. 9b,c,d at 101 and 436 cm^−1^ respectively^41^. The nonpolar modes (E2) are Raman active and have two frequencies, E2 (high) and E2 (low), associated with the vibration of the oxygen atom and vibration of Zn atoms, The peak at 101 cm^−1^ represents the E2_(Low)_ (E_21_) mode, and the peak at 360 cm^−1^ represents the E2_(High)_ (E2_h_) mode^42^.

#### Particle size distribution

**Figure 10:**
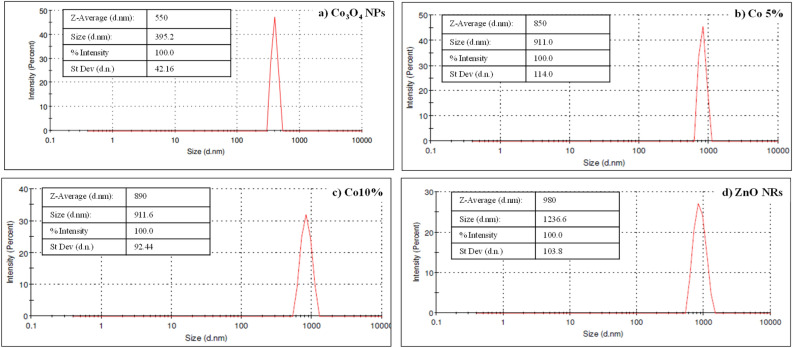
DLS particle size distribution of (**a**) Co_3_O_4_ NPs, (**b**) Co 5%, (**c**) Co 10% and (**d**) ZnO NRs.

Dynamic light scattering (DLS) can be used to determine the hydrodynamic diameter of produced nanoparticles, nanorods, and nanocomposites. Figure 10 shows the DLS, which reveals the hydrodynamic diameter of the nanomaterials.

When light passes through a colloidal solution, it bombards microscopic particles and scatters them in every way possible (i.e. Rayleigh scattering). Even if the incident light is monochromatic or laser, we see a fluctuation in the intensity of light. This fluctuation in light intensity is caused by Brownian motion in solution, which is constantly occurring. DLS, also known as photon correlation scattering, is a common name for this approach.

The average particle size of biogenic Co_3_O_4_ NPs (Fig. 10a) was 729 nm, according to DLS. Biogenic Co_3_O_4_ NPs have a strong peak, indicating their mono-dispersed nature. Figure 10d shows the distribution of ZnO NRs by size, which ranges from 1000 to 3000 nm. ZnO NRs have an average particle size of 1733 nm. The poly-dispersed nature of ZnO Nanorods can be seen in their broad size distributions. Figure 10b,c show the particle size distribution of nanocomposites. Ball milling lowers the particle size of both nanocomposites when compared to ZnO NRs. The particle size of Co 5% is 810 nm. Co 10% has a diameter of 1156 nm. Both nanocomposites are polydispersed^43^.

#### Electro kinetic potential and zeta potential

To find out the stability of synthesized nanomaterial, the electrokinetic potential was used. Additionally, it also sheds light on the dispersion stability of colloidal solutions and the mobility of nanoparticles as well. The material is more stable as the value of zeta potential, either positive or negative, is higher.

**Figure 11:**
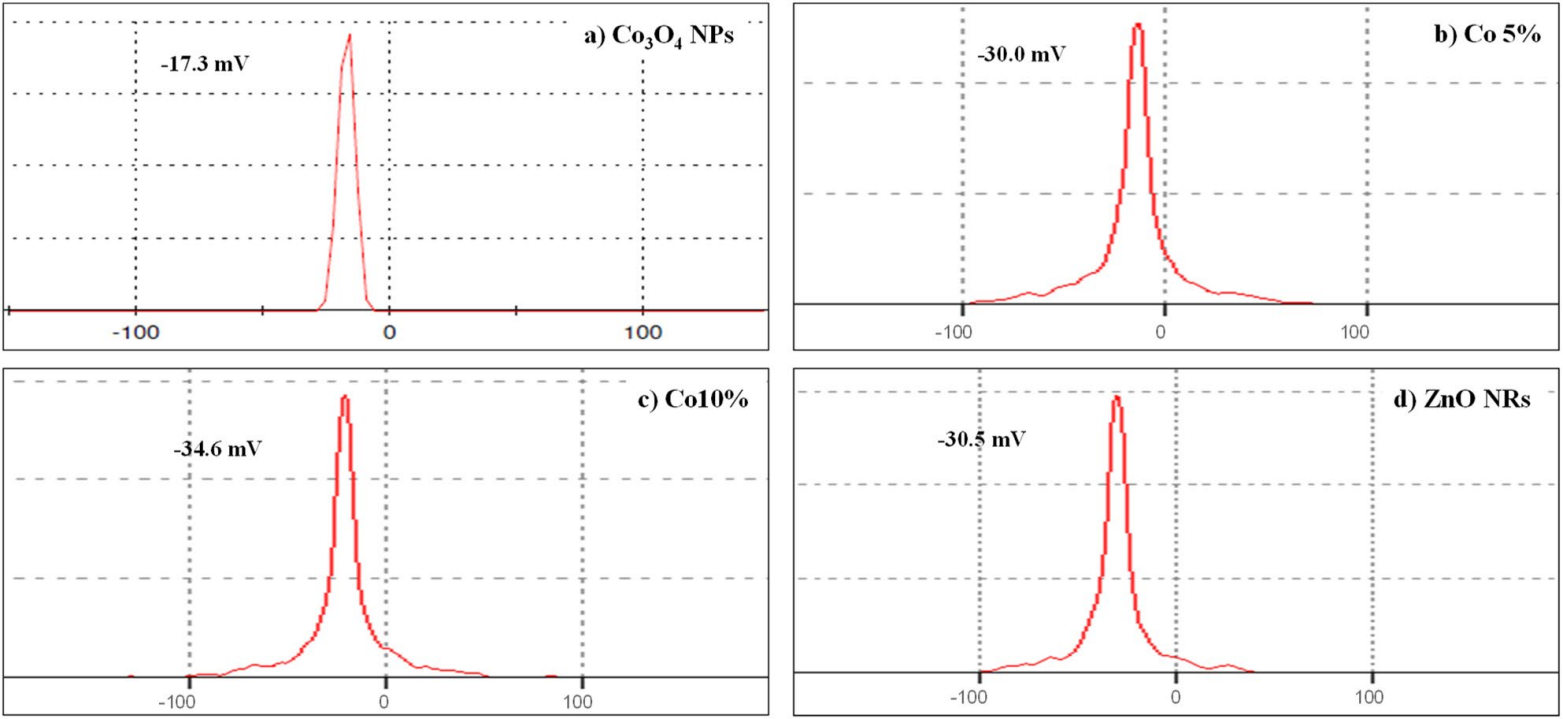
Zeta potential distribution of (**a**) Co_3_O_4_ NPs, (**b**) Co 5%, (**c**) Co 10% and (**d**) ZnO NRs.

The zeta potential of Co_3_O_4_ NPs is shown in Fig. 11a, and it is − 17.3 mV. Furthermore, looking at Fig. 11d, ZnO NRs show a zeta potential value of − 30.5 mV. The high negative zeta potential (ξ) value supports the long-term stability, good colloidal nature, and high dispersion of ZnO NRs due to negative-negative repulsion. Figure 11b,c illustrate the zeta potential values of Co 5% and Co 10%, and the values are − 30.0 mV and − 34.6 mV respectively. The synthesized nanomaterials have zeta potential values of between − 35 and 0 mV and have outstanding stability. Nevertheless, the dispersion stability also affects the zeta potential values^44,45^.

#### Thermogravimetric analysis

**Figure 12:**
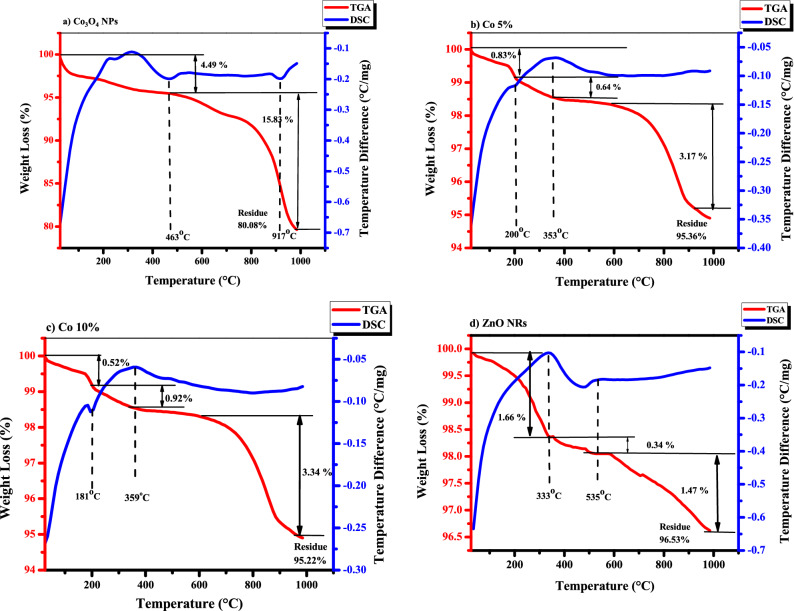
TGA and DSC analysis of (**a**) Co_3_O_4_ NPs, (**b**) Co 5%, (**c**) Co 10% and (**d**) ZnO NRs.

Figure 12a–d depicts typical TGA/DSC curves of biosynthesized Co_3_O_4_ NPs, ZnO NRs, and their nanocomposites at a heating rate of 10 °C/min. The TGA profile of Co_3_O_4_ NPs (Fig. 12a), which show a larger weight loss than others, shows a steady weight reduction with two quasi-sharp shifts at 463 °C and 917 °C, followed by a practically constant plateau. The solvent is to blame for the weight loss. The evaporation of water molecules and nitrogen causes a 4.49% weight loss from 100 to 463 °C. Nitrogen loss leading to nitrate breakdown causes the peak at 463 °C. However, due to the phase change of the material, there is a weight loss of 15.83% between 463 and 1000 °C^28,46^.

When it comes to ZnO NRs (Fig. 12d), annealing at a temperature of over 300 °C appears to ensure the production of stable ZnO NRs. The loss of volatile surfactant molecules adsorbed on the surface of Zn complexes during synthesis conditions might account for the weight of up to 300 °C. The conversion the of Zn complex to zinc hydroxide is responsible for the exothermic peak of about 333 °C. The creation of ZnO NRs and the degradation of organic molecules might be attributed to the 2nd exothermic peak at 535 °C. A 96.53% residual is left after the last degradation, which takes place at 700 °C and produces ZnO with a wurtzite-like structure that is stable up to 1000 °C^47^. In short, ZnO NRs were more thermally stable as compared to the Co_3_O_4_ NPs.

Co 5% and Co 10% (Fig. 12b,c) show relevantly similar results, with the two endothermic peaks. The 1st peak is at 200 °C and 181 °C, respectively. These peaks are due to the different weight variations of the Co_3_O_4_ NPs and ZnO NRs. Moreover, the peaks at 353 °C and 359 °C discretely were present because of the conversion of the zinc hydroxide into zinc oxide. Finally, both samples left 95.36% and 95.22% of residue, respectively. Finally, both samples left 95.36% and 95.22% of residue, respectively^48^.

#### Determination of antimicrobial activities of Co_3_O_4_ NPs, ZnO NRs, and nanocomposite (Co 5% and Co 10%)

The MIC of Co_3_O_4_ NPs, ZnO NRs, and their nanocomposites were studied by turbidity measurement using a spectrophotometric method at 625 nm^49^. At lower concentrations of 50 to 200 μg/mL, no visible growth was observed under a spectrophotometer, indicating that this concentration has a strong bactericidal action, which is essential in the manufacture of antibacterial compounds. The existence or absence of turbidity, which was evaluated by + or − in Table 3, was demonstrated. Because of bacterial growth, lower concentrations of nanomaterials appear turbid. This suggests that NPs at lower concentrations have minimal antibacterial effects. The results also show that the combination of 90% ZnO + 10% Co_3_O_4_ has the highest potential bactericidal activity of any nanomaterial sample. The results also showed that bacterial strains like *Staphylococcus aureus* and *Salmonella typhi* were extremely susceptible to nanomaterials, but *Bacillus cereus* and *Escherichia coli* had reduced bactericidal efficacy as seen by observable growth. Previous studies by Raj et al.^50^ demonstrated the MIC of zinc nanoparticles prepared from Brassica *oleraceae* leaves against similar types of bacteria.

**Table 3:** Tabular representation of bacterial growth observed in different concentrations of ZnO NRs, Co_3_O_4_ NPs, Co 10%, and Co 5% nanocomposite after 24 h.

Sample name/strain name	*Conc* µg/mL	*Escherichia coli* (NCIM 2662)	*Salmonella typhi* (NCIM 5278)	*Bacillus cereus* (NCIM 2217)	*Staphylococcus aureus* (NCIM 5276)
ZnO NRs	25	+	+	+	+
	50	+	−	+	−
	100	−	−	−	−
	150	−	−	−	−
	200	−	−	−	−
Co_3_O_4_ NPs	25	+	+	+	+
	50	+	−	+	−
	100	−	−	−	−
	150	−	−	−	−
	200	−	−	−	−
Co 10%	25	+	−	+	+
	50	+	−	+	−
	100	−	−	−	−
	150	−	−	−	−
	200	−	−	−	−
Co 5%	25	+	−	+	+
	50	−	−	−	−
	100	−	−	−	−
	150	−	−	−	−
	200	−	−	−	−
Streptomycin	50	−	−	−	−
Negative control	NA	+	+	+	+

Positive (+): turbidity in the medium due to growth of microbes, negative (−): turbidity does not occur in the medium due to no visible growth. NA- does not contain any sample.

An earlier report on different methodologies for nanoparticle preparation and application from cow urine was detailed and discussed by Dabhane et al.^51^. Previous literature on structural properties of ZnO NRs and antibacterial proficiency based on four mechanisms for the production of reactive oxygen species (ROS) were studied by Bruna Lallo da Silva et al.^52^ in a review study, which is similar to the current study.

These antibacterial and antifungal strategies for nanomaterials prepared were assessed against a set of four bacterial and two fungal strains shown in Table 4. The presence or absence of inhibition zones in mm was used to measure potency qualitatively. The observations are represented in Table 4 and indicate that the concentrations of 200 µg/mL nanomaterials extract show higher significant antimicrobial activity against all gram-negative bacterial strains and are depicted in tabular form in Table 4. The zone of inhibition observed is 17 ± 0.81 mm for the microbe *S. aureus* which is concluded to be better activity than similar ZnO NRs using solanum nigrum leaf extract in both Gram-positive (*S. aureus*) and Gram-negative (*S. paratyphi, V. cholera, E. coli*) bacteria were studied by Ramesh et al.^53^. In *A. niger* the minor antimicrobial spectrum of the inhibition zone was found (15.66 ± 0.94) than *Fusarium solani* (14.66 ± 0.47) which indicates the highest antifungal activity in the Co 5% sample. A result clearly shows that the combination method has more advantages than another singular metallic cow urine nanoparticle preparation method.

**Table 4:** Anti-bacterial and anti-fungal effect exhibited by ZnO NRs, Co_3_O_4_ NPs, Co 10%, and Co 5% nanocomposite on various test microbes (values in nm) (mean ± SD).

Sample name/strain name	*Conc* µg/mL	Bacterial strains			Fungal stains		
		*Escherichia coli*	*Salmonella typhi*	*Bacillus cereus*	*Staphylococcus aureus*	*Aspergillus niger*	*Fusarium solani*
ZnO NRs	50	11.33 ± 0.47	12.66 ± 0.94	13.33 ± 0.47	12.66 ± 0.94	12 ± 0.81	10.33 ± 0.47
	100	11.66 ± 0.47	14.66 ± 0.47	13.66 ± 0.47	13.33 ± 0.47	12.33 ± 1.24	11.66 ± 0.94
	150	14.33 ± 0.47	15.00 ± 0	15.33 ± 0.47	15.33 ± 0.47	12.66 ± 0.47	12 ± 0.81
	200	15.66 ± 0.47	16.66 ± 0.47	15.01 ± 0	16.33 ± 0.47	14.33 ± 0.97	13.66 ± 0.47
Co_3_O_4_ NPs	50	10.33 ± 0.47	12.33 ± 0.47	13.07 ± 0.81	12.0 ± 0.81	12 ± 0.81	11.33 ± 0.47
	100	13 ± 0	14.66 ± 0.47	13.33 ± 0.47	12.33 ± 0.47	12.33 ± 0.47	12 ± 0
	150	12.66 ± 0.47	15.33 ± 0.47	13.33 ± 0.47	13.33 ± 0.47	13 ± 0.81	12.66 ± 0.47
	200	14.66 ± 0.47	15.66 ± 0.47	14 ± 0.81	14.33 ± 0.47	13.66 ± 0.47	13.33 ± 0.47
Co 10%	50	11.66 ± 0.47	13.33 ± 0.47	14 ± 0.81	14.66 ± 0.47	13.33 ± 0.47	11.66 ± 0.47
	100	12.66 ± 0.47	14.33 ± 0.47	15 ± 0.81	15.66 ± 0.47	14.33 ± 0.47	12.33 ± 0.47
	150	15 ± 0	15.33 ± 0.47	15.33 ± 0.47	15.33 ± 0.47	15 ± 0.81	13.66 ± 0.47
	200	16.33 ± 0.47	16.33 ± 0.47	16.33 ± 0.47	17 ± 0.81	15.66 ± 0.94	14.66 ± 0.47
Co 5%	50	11.66 ± 0.94	12.66 ± 0.47	14 ± 0.81	12.66 ± 0.47	11.66 ± 0.47	11.66 ± 0.47
	100	12.66 ± 0.47	13.33 ± 0.47	15.66 ± 0.47	14.66 ± 0.47	13 ± 0.81	11.33 ± 0.47
	150	14.33 ± 0.47	14.33 ± 0.47	14.66 ± 0.94	15.66 ± 0.47	13 ± 0.81	12.33 ± 0.47
	200	15.33 ± 0.47	15.33 ± 0.81	16 ± 0.81	16.66 ± 0.94	14.66 ± 0.47	12.66 ± 0.47
Streptomycin	50	17.66 ± 0.47	17 ± 0.81	17 ± 0.81	18.66 ± 0.47	15.33 ± 0.47	15.66 ± 0.47

#### Antioxidant activity

**Figure 13:**
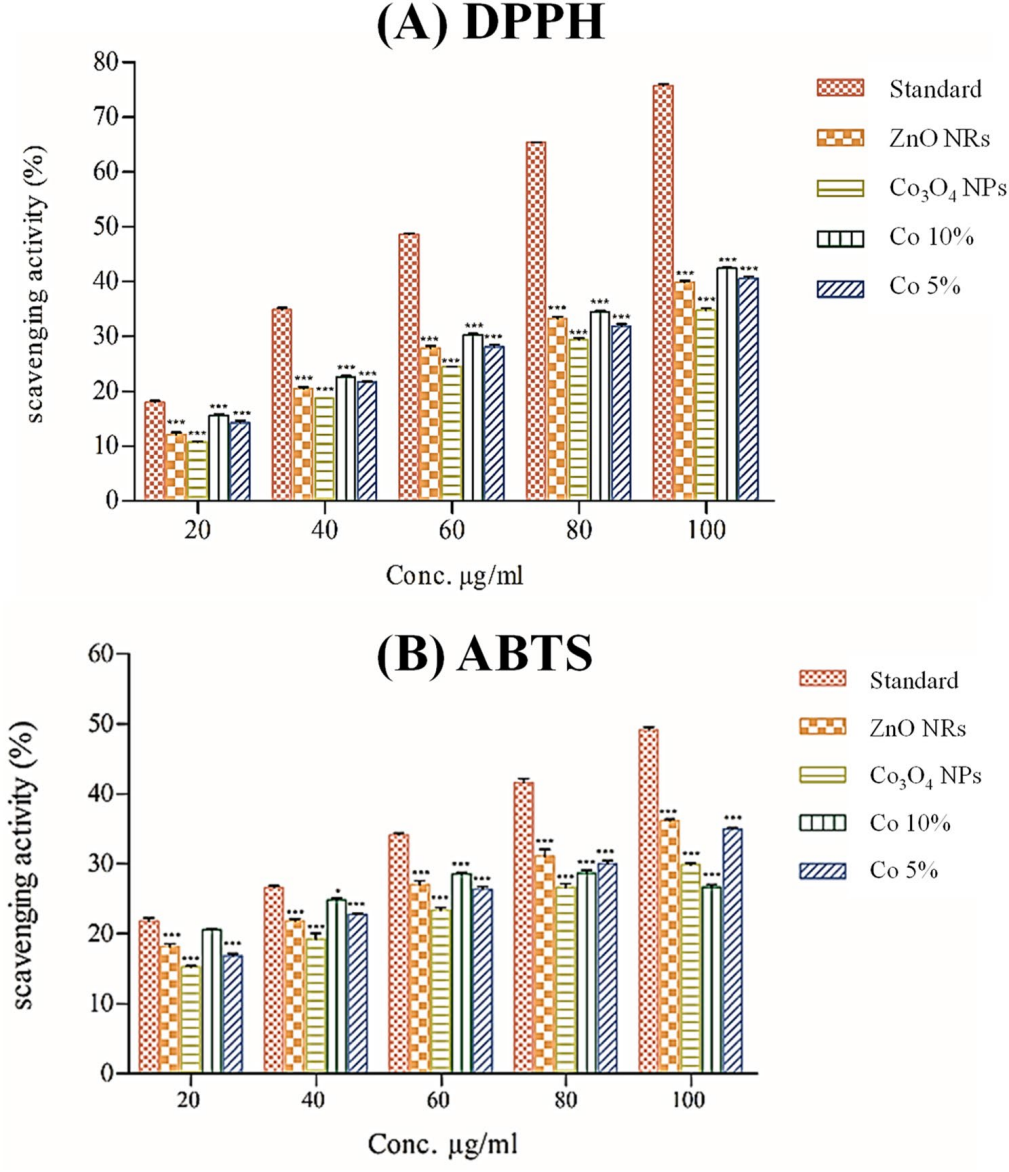
Comparative study antioxidant of activity using (**A**) DPPH and (**B**) ABTS radical scavenging potential with different concentrations of Standard, ZnO NRs, Co_3_O_4_ NPs, Co 10% and Co 5% nanocomposite. (n = 3). Data represent the mean ± SD; N = 3. *P < 0.01, **P < 0.001, ***P < 0.0001 compared with control.

Antioxidants are free radical molecules that are created by a variety of systems that have the potential to harm biological cellular processes. ABTS and DPPH are two methods that are often used for measuring free radical destructive activity^54^. The DPPH and ABTS scavenging activities of nanomaterials are shown in Fig. 13 in comparison to standard antioxidant ascorbic acid, indicating maximum antioxidant activity, whereas the cumulative effect of Co_3_O_4_ NPs shows maximum potential, which is 42.41 ± 0.18% in DPPH radical savaging activity and 42.41 ± 0.18% in ABTS radical scavenging activity at 100 µg/mL, whereas standard ascorbic acid shows 75.68 ± 0.47% activity.

The dark violet color of the DPPH was gradually decreasing over a time interval, and a decrease in absorbance was also recorded. The decrease in absorption intensity confirms DPPH's good scavenging activities, which are due to its ability to be a good oxidant, electron-losing, and capping agent on the surface of various nanomaterials. Our results show similar contact with Ag_2_O and ZnO NRs using cow urine have different applications in photoluminescence, photolytic, antibacterial, and antioxidant activities as reported previously by Vinay et al.^55^ and Dabhane et al.^51^. Significantly, the biogenic synthesis of Co_3_O_4_ NPs and the hydrothermal synthesis of ZnO NRs exhibit a broad spectrum of antibacterial and antioxidant activity. As a result, it signifies promising antioxidants and antibacterial agents with potential use in the synthesis of pharmaceutical drugs. The order of maximum DPPH potential and ABTS potential is Co 10% ˃ Co 5% ˃ ZnO NRs ˃ Co_3_O_4_ NPs and Co 5% ˃ZnO NRs ˃ Co 10% ˃ Co_3_O_4_ NPs respectively. The order of value is 42.41 ± 0.18 ˃ 40.57 ± 0.58 ˃ 39.88 ± 0.43 ˃ 34.88 ± 0.48 and 36.19 ± 0.25 ˃ 34.97 ± 0.28 ˃ 29.89 ± 0.35 ˃ 26.66 ± 0.47.

#### Anti-inflammatory study

**Figure 14:**
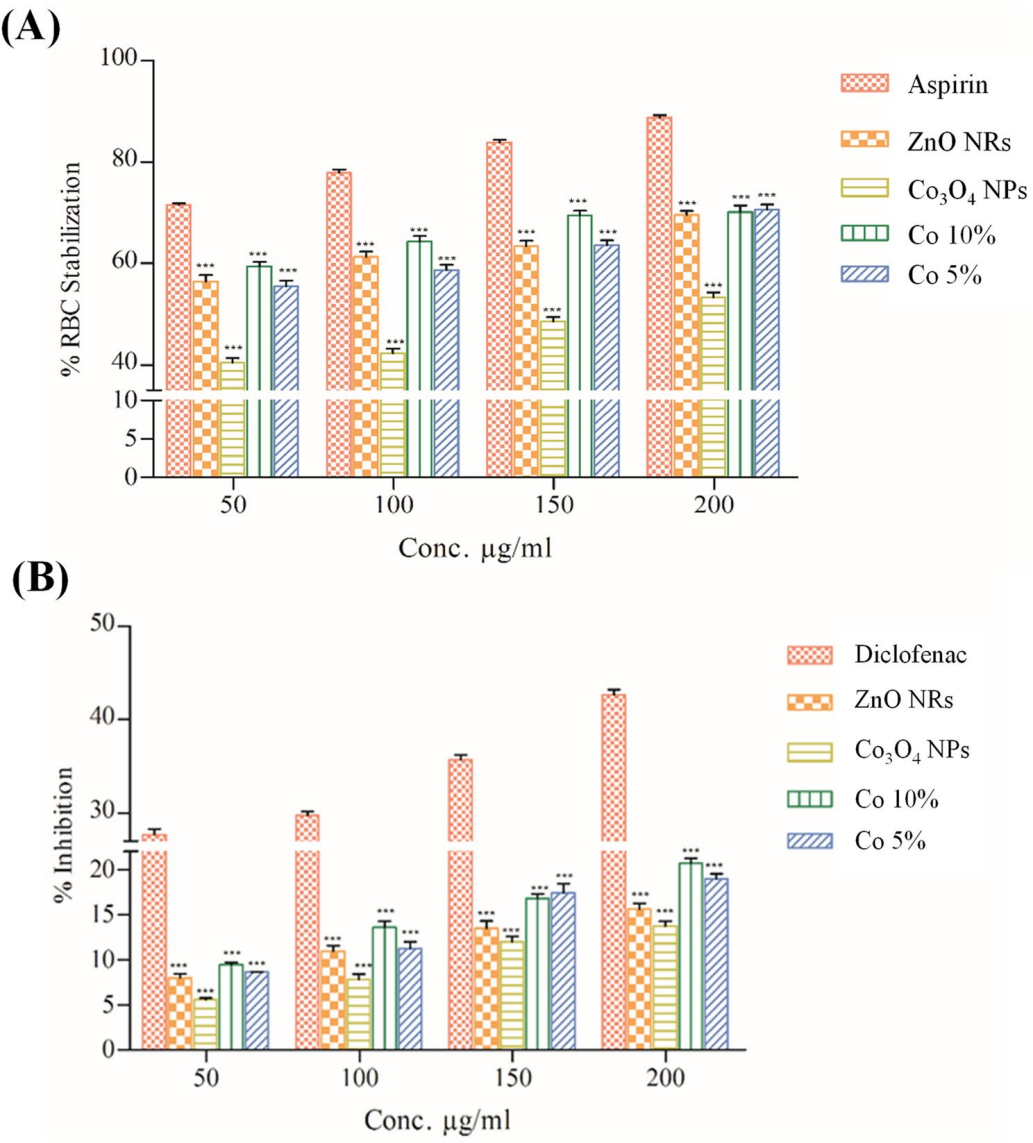
Anti-inflammatory effects of ZnO NRs, Co_3_O_4_ NPs, Co 10%, and Co 5% nanocomposite assessed against (**A**) Heat-induced egg albumin denaturation and **(B**) RBC Stabilization of Leukocyte (%). Data represent the mean ± SD; N = 3. *P < 0.01, **P < 0.001, ***P < 0.0001 compared with control.

Previous literature by Agarwal et al.^56^ clearly determines the mechanism-based anti-inflammatory properties of the NPs from several metals and metal oxides. NPs have promising anti-inflammatory properties due to their large surface area to volume ratio, which will be better at blocking inflammation enhancers e.g. cytokines and inflammation-assisting enzymes. The in vitro assessment of BSA denaturation potential, which results in anti-inflammatory effects of nanoparticles assessed against heat-induced egg albumin denaturation, is summarized in Fig. 14. In a concentration-dependent manner, all tested doses effectively inhibited the denaturation of egg albumin. Whereas the maximum BSA Denaturation % inhibition 7 of 3.53 ± 0.14% was observed at the highest concentration of Co 10%of 200 μg/mL. The order of maximum shown below is Co 10% ˃ Co 5% ˃ ZnO NRs ˃ Co_3_O_4_ NPs and values are 67.46% ˃ 63.03%˃ 66.40% ˃59.04%. At the concentration of 50 μg/mL, aspirin, used as a standard drug, inhibited the enzyme by 61.91 ± 0.24%.

RBC Stabilization of Leukocyte is one of the methods used to measure inflammatory response by measuring the hemoglobin absorbance spectrophotometrically at 560 nm. Anti-inflammatory drugs may lyse and usually cause lymphocyte reorganization, resulting in a rapid decrease in the number of lymphocytes in the peripheral blood, which causes a longer-term response. Because the erythrocyte membrane is comparable to the lysosomal membrane, the HRBC technique was chosen for in vitro assessment of anti-inflammatory efficacy. Its stabilization means that the NPs may just as well stabilize lysosomal membranes. Results demonstrated in Fig. 14 indicate that water extract of Co 10% solution has noteworthy anti-inflammatory action at various concentrations. Whereas ZnO NRs and Co_3_O_4_ NPs separately provide lower RBC Stabilization of Leukocyte (%) values of 15.15 ± 0.24% and 13.65 ± 0.24%, Respectively at a concentration of 200 μg/mL. Whereas Co 5% had a slightly lower anti-inflammatory potential than Co 10%, and standard drug diclofenac had a potential of 27.52 ± 0.94%, as shown in Fig. 14. The given results are similar to previous literature on anti-inflammatory potential and antioxidant of zinc oxide nanoparticles synthesized using Polygala tenuifolia root extract^57^ further studies of anti-inflammatory and antinociceptive activities in the mice model were explained by Liu et al.^58^.

## Conclusions

The results confirm that the biomolecules present in the physiologically processed liquid metabolic waste of Indian cows are responsible for the successful formation of cobalt oxide nanomaterials. When we analyze these materials, we discover that they have unique characterization results. We have the composite's conformation by XRD spectra and EDX analysis. Because of the low zeta potential value, the morphology of Co_3_O_4_ NPs is aggregation form as compared to others. This suggests that the substance isn't very stable. But the stability is increased by making a composite of ZnO and Co_3_O_4_. In FTIR, we observed that both Co^2+^ and Co^3+^ species are present in our material, as well as the conformation of the Zn–O bond. The results demonstrated an inexpensive, simple, and eco-friendly method for synthesizing Co_3_O_4_ NPs, ZnO NRs, and their composites, which verified excellent antioxidant, antimicrobial, and anti-inflammatory activities. In addition to that, the nanocomposite shows excellent antioxidant and anti-inflammatory properties as compared to the pure nanomaterial. Look into the details, according to ABTS and DPPH methods Co 10% and Co 5% exhibit high antioxidant potential respectively. Nevertheless, as compared to others the Co 10% shows greater anti-inflammatory potential as well as bactericidal activity potential. Moreover, all these nanomaterials are vulnerable to *S. aureus* and *S. typhi.* Thus, we believe that all of these new nanomaterials should be considered as possible drugs for the management and treatment of various disorders.

## Data Availability

The data presented in this study are available on request from the corresponding author.

## References

[1] Li H (2019). Zinc cobalt sul fi de nanoparticles as high performance electrode material for asymmetric supercapacitor. Electrochim. Acta.

[2] Rasheed T (2019). Carbon nanotubes-based cues: A pathway to future sensing and detection of hazardous pollutants. J. Mol. Liq..

[3] Karvekar OS, Sarvalkar PD, Vadanagekar AS, Singhan RD, Jadhav SM (2022). Biogenic synthesis of silver anchored ZnO nanorods as nano catalyst for organic transformation reactions and dye degradation. Appl. Nanosci..

[4] Patil AA (2020). Bipolar-resistive switching and memristive properties of solution—Processable cobalt oxide nanoparticles. J. Mater. Sci. Mater. Electron..

[5] Wang X (2021). Construction of cobalt nanoparticles decorated intertwined N-doped carbon nanotube clusters with dual active sites for highly effective 4-nitrophenol reduction. J. Alloys Compd..

[6] Sarvalkar PD (2021). Bio-mimetic synthesis of catalytically active nano-silver using *Bos taurus* (A-2) urine. Sci. Rep..

[7] Waris A (2021). Green fabrication of Co and Co_3_O_4_ nanoparticles and their biomedical applications: A review. Open Life Sci..

[8] Khezri K, Saeedi M, Maleki S (2018). Biomedicine and pharmacotherapy application of nanoparticles in percutaneous delivery of active ingredients in cosmetic preparations. Biomed. Pharmacother..

[9] Sarvalkar, P. D. *et al.* A review on multifunctional nanotechnological aspects in modern textile. *J. Text. Inst.* 1–18 (2022).

[10] Prasad RD (2021). A review on concept of nanotechnology in veterinary medicine. ES Food Agrofor..

[11] Jadoun S, Arif R, Jangid NK, Meena RK (2021). Green synthesis of nanoparticles using plant extracts: A review. Environ. Chem. Lett..

[12] Meena M (2019). Go mutra (Cow urine) and its uses: An overview. J. Entomol. Zool. Stud..

[13] Nazeruddin GM (2017). *Bos taurus* urine assisted synthesis of cadmium nanoparticles. DER PHARMA Chem..

[14] Padvi SRPMN, Shaikh SSSYI, Samant LSCAP, Prasad NR (2020). Bio-inspired synthesis of catalytically and biologically active palladium nanoparticles using *Bos taurus* urine. SN Appl. Sci..

[15] Padvi MN (2020). *Bos taurus* urine assisted biosynthesis of CuO nanomaterials: A new paradigm of antimicrobial and antineoplatic therapy. Macromol. Symp..

[16] Kamble P, Suryawanshi S, Jadhav JP, Attar YC (2019). Enhanced inulinase production by *Fusarium solani* JALPK from invasive weed using response surface methodology. J. Microbiol. Methods.

[17] Mohanty I, Senapati MR, Jena D (2014). Diversified uses of cow dung. Int. J. Pharm. Pharm. Sci..

[18] Dastager SG, Sreedhar B, Dayanand A, Shirley AD (2010). Antimicrobial activity of silver nanoparticles synthesized from novel streptomyces species. Dig. J. Nanomater. Biostruct..

[19] Suryawanshi SS, Rane MRS (2019). Antioxidant, antimicrobial activity with mineral composition and LCMS based phytochemical evaluation of some Mucuna species from India. Int. J. Pharm. Biol. Sci..

[20] Re R (1999). Antioxidant activity applying an improved ABTS radical. Free Radic. Biol. Med..

[21] Jadhav P (2020). Green AgNPs decorated ZnO nanocomposites for dye degradation and antimicrobial applications. Eng. Sci..

[22] Brand-Williams W, Cuvelier ME, Berset C (1995). Use of a free radical method to evaluate antioxidant activity. LWT Food Sci. Technol..

[23] Suryawanshi S, Kshirsagar P, Kamble P, Bapat V, Jadhav J (2022). Systematic enhancement of l-DOPA and secondary metabolites from *Mucuna imbricata*: Implication of precursors and elicitors in Callus culture. S. Afr. J. Bot..

[24] Grant NH, Alburn HE, Kryzanauskas C (1970). Stabilization of serum albumin by anti-inflammatory drugs. Biochem. Pharmacol..

[25] Rane M, Suryawanshi R (2019). Exploring the proximate composition, antioxidant, anti-Parkinson’s and anti-inflammatory potential of two neglected and underutilized Mucuna species from India. S. Afr. J. Bot..

[26] Bhurat M, More S, Sanghavi R, Salunkhe P, Umarkar A (2011). Preclinical evaluation of *Remusatia Vivipara* leaves extracts on haloperidol induced catalepsy in experimental animals. Int. J. Pharm. Biol. Sci..

[27] Aware C (2017). Evaluation of l-dopa, proximate composition with in vitro anti-inflammatory and antioxidant activity of *Mucuna macrocarpa* beans: A future drug for Parkinson treatment. Asian Pac. J. Trop. Biomed..

[28] Akhlaghi N, Najafpour-darzi G, Younesi H (2020). Facile and green synthesis of cobalt oxide nanoparticles using ethanolic extract of *Trigonella foenumgraceum* (Fenugreek) leaves. Adv. Powder Technol..

[29] Suryavanshi RD (2018). Nanocrystalline immobilised ZnO photocatalyst for degradation of benzoic acid and methyl blue dye. Mater. Res. Bull..

[30] Theivasanthi, T. & Alagar, M. Titanium dioxide (TiO_2_) nanoparticles XRD analyses: An insight. http://arxiv.org/abs/1307.1091 (2013).

[31] Huang N (2015). One-step pyrolytic synthesis of ZnO nanorods with enhanced photocatalytic activity and high photostability under visible light and UV light irradiation. J. Alloys Compd..

[32] Kumar R (2021). Spindle-like Co_3_O_4_–ZnO nanocomposites scaffold for hydrazine sensing and photocatalytic degradation of rhodamine B dye. Eng. Sci..

[33] Bhat DK (2008). Facile synthesis of ZnO nanorods by microwave irradiation of zinc–hydrazine hydrate complex. Nanoscale Res. Lett..

[34] Yi SH, Choi SK, Jang JM, Kim JA, Jung WG (2007). Low-temperature growth of ZnO nanorods by chemical bath deposition. J. Colloid Interface Sci..

[35] Deepty M (2019). XRD, EDX, FTIR and ESR spectroscopic studies of co-precipitated Mn-substituted Zn–ferrite nanoparticles. Ceram. Int..

[36] Hassanpour M, Safardoust-hojaghan H, Salavati-niasari M (2016). Degradation of methylene blue and Rhodamine B as water pollutants via green synthesized Co_3_O_4_/ZnO nanocomposite. J. Mol. Liq..

[37] Cruz JC (2019). Synthesis and characterization of cobalt nanoparticles for application in the removal of textile dye. J. Environ. Manag..

[38] Abdallah AM, Awad R (2021). Sm and Er partial alternatives of Co in Co_3_O_4_ nanoparticles: Probing the physical properties. Phys. B Phys. Condens. Matter.

[39] Beura R, Pachaiappan R, Paramasivam T (2021). Photocatalytic degradation studies of organic dyes over novel Ag-loaded ZnO–graphene hybrid nanocomposites. J. Phys. Chem. Solids.

[40] Chanda A (2017). Study of structural, optical and magnetic properties of cobalt doped ZnO nanorods. RSC Adv..

[41] Phan T (2012). Enhancement of multiple-phonon resonant Raman scattering in Co-doped ZnO nanorods. Appl. Phys. Lett..

[42] Had B (2016). Laser power influence on Raman spectra of ZnO (Co) nanoparticles. J. Phys. Chem. Solids.

[43] Fan L (2007). Hydrothermal synthesis and photoluminescent properties of ZnO nanorods. J. Lumin..

[44] Tang L (2014). Cobalt nanoparticles-embedded magnetic ordered mesoporous carbon for highly effective adsorption of rhodamine B. Appl. Surf. Sci..

[45] Marsalek R (2014). Particle size and zeta potential of ZnO. APCBEE Proc..

[46] Nouroozi F, Farzaneh F (2011). Synthesis and characterization of brush-like ZnO nanorods using albumen as biotemplate. J. Braz. Chem. Soc..

[47] Zhou N (2017). Synthesis and characterization of Zn_1__−__x_Co_x_O green pigments with low content cobalt oxide. J. Alloys Compd..

[48] Xu C, Wang X, Zhu J, Yang X, Lu L (2008). Deposition of Co_3_O_4_ nanoparticles onto exfoliated graphite oxide sheets. J. Mater. Chem..

[49] Pfaller MA, Messer SA, Coffmann S (1995). Comparison of visual and spectrophotometric methods of MIC endpoint determinations by using broth microdilution methods to test five antifungal agents, including the new triazole D0870. J. Clin. Microbiol..

[50] Raj A, Lawrence RS, Jalees M, Lawrence K (2015). Anti-bacterial activity of zinc oxide nanoparticles prepared from *Brassica oleraceae* leaves extract. Int. J. Adv. Res..

[51] Dabhane H, Zate M, Bharsat R, Jadhav G, Medhane V (2021). A novel bio-fabrication of ZnO nanoparticles using cow urine and study of their photocatalytic, antibacterial and antioxidant activities. Inorg. Chem. Commun..

[52] da Silva BL (2019). Relationship between structure and antimicrobial activity of zinc oxide nanoparticles: An overview. Int. J. Nanomed..

[53] Ramesh M, Anbuvannan M, Viruthagiri G (2015). Green synthesis of ZnO nanoparticles using *Solanum nigrum* leaf extract and their antibacterial activity. Spectrochim. Acta Part A Mol. Biomol. Spectrosc..

[54] Bartkowiak A, Roy S (2022). Alginate biofunctional films modified with melanin from watermelon seeds and zinc oxide/silver nanoparticles. Materials (Basel).

[55] Vinay SP, Udayabhanu, Nagaraju G, Chandrappa CP, Chandrasekhar N (2019). Novel Gomutra (cow urine) mediated synthesis of silver oxide nanoparticles and their enhanced photocatalytic, photoluminescence and antibacterial studies. J. Sci. Adv. Mater. Devices.

[56] Agarwal H, Nakara A, Shanmugam VK (2019). Anti-inflammatory mechanism of various metal and metal oxide nanoparticles synthesized using plant extracts: A review. Biomed. Pharmacother..

[57] Nagajyothi PC (2015). Antioxidant and anti-inflammatory activities of zinc oxide nanoparticles synthesized using *Polygala tenuifolia* root extract. J. Photochem. Photobiol. B.

[58] Liu H (2020). Zinc oxide nanoparticles synthesised from the *Vernonia amygdalina* shows the anti-inflammatory and antinociceptive activities in the mice model. Artif. Cells Nanomed. Biotechnol..

